# RNA editing: an emerging frontier in cancer therapy – explorations, opportunities, and challenges

**DOI:** 10.1097/JS9.0000000000004547

**Published:** 2026-01-12

**Authors:** Jun Chen, Qi Sun, Na Gong, Zengkan Du, Zhijie Zhao, Dongyang Ding, Haoling Zhang, Qilu Yan, Shengxian Yuan, Shuya Jiang

**Affiliations:** a The Third Department of Hepatic Surgery, Eastern Hepatobiliary Surgery Hospital, Naval Medical University, Shanghai, China; b School of Basic Medicine, Naval Medical University, Shanghai, China; c Shandong University of Traditional Chinese Medicine, Jinan, China; d Department of Nephrology, First Medical Center of Chinese PLA General Hospital, Beijing, China; e Department of Gastroenterology, National Clinical Research Center for Digestive Diseases, Changhai Hospital, Naval Medical University, Shanghai, China; f Department of Plastic and Reconstructive Surgery, Shanghai Ninth People’s Hospital, Shanghai JiaoTong University School of Medicine, Shanghai, China; g Department of Biomedical Sciences, Advanced Medical and Dental Institute, Universiti Sains Malaysia, Kepala Batas, Penang, Malaysia; h Cancer Center, Renmin Hospital of Wuhan University, Wuhan, Hubei, China

**Keywords:** ADAR, APOBEC, A-to-I editing, cancer, C-to-U editing, RNA editing

## Abstract

RNA editing is a critical regulatory mechanism that acts after transcription. Two types of RNA editing are A-to-I editing and C-to-U editing. The first one uses adenosine deaminase acting on RNA, while the second uses APOBEC complexes. RNA editing has an important role in cancer development in controls tumorigenesis and tumor progression through multiple mechanism. It allows for precise edits to RNA sites linked to cancer at a molecular level. At the microenvironmental level, it regulates the immune response by remodeling the immune landscape. RNA editing has dual roles in tumor progression and anti-tumor effects. The ability of some non-coding RNAs to perform the reverse function is shown to be useful in various important biological processes. The important processes include drug resistance, agitation, tumor suppressor gene expression, tumor microenvironment, metabolic regulation, and cancer cell migration. Both the over and under editing of RNA can cause tumors. Therefore, initiating and stopping editing needs to be strictly controlled for clinical purposes. Studies indicate RNA editing has a unique therapeutic potential by downregulating oncogenes, restoring mutated genes, and enhancing the efficacies of immunotherapy. Problems remain; however, with regard to targeting precision, safety, functional characterization, and the regulation of editing efficiency and extent. The ongoing evolution of specificity and tools may ensure that RNA editing emerges as a vital strategy in cancer therapeutics. Nevertheless, progress is hindered by enduring hurdles, such as off-target effects and variable editing efficiency, and has yet to be validated in larger and multi-site clinical trials. In the same vein, RNA editing may serve as useful references for surgical decisions. For example, RNA editing can be used to identify surgery risk, recurrence risk, and surgical approach selection due to its exact targeting capacity. It also helps intraoperative resection margins and adjuvant therapy to properly maintain the outcome postoperatively in the long term. Hence, surgical oncology researchers must explore RNA editing in-depth. It is worth noting that limited antitumor activity was observed in solid tumors in preclinical studies due to insufficient delivery of editing tools, indicating realistic translation expectation is needed.

## Introduction

RNA editing is a post-transcriptional modification that changes the sequences of RNA. The phenomenon was first discovered in 1986 by Benne and co-workers studying the mitochondrial cytochrome oxidase gene in Trypanosoma^[1]^. The development of next-generation sequencing has drastically sped up the identification of more edited RNA sites in species^[2]^. Studies suggest that RNA editing may increase the ability of prostate, liver, and colon cancers to invade other tissues^[3,4]^. In contrast, RNA editing in gastric cancer, melanoma, and breast cancer appears to inhibit tumor development^[5,6]^. These results may offer new avenues for research and clinical interventions based on these newly discovered insights into tumor pathogenesis.

In mammals, RNA editing significantly occurs in two types, namely A-to-I and C-to-U editing^[7]^. The most common kind of A-to-I editing is generally resulted due to adenosine deaminase acting on RNA (ADAR). Moreover, ADAR hydrolizes deamination in the greater numbers of non-coding sequences^[7,8]^. Within the deaminase domain, ADAR acts on conserved residues critical for the catalytic process, including zinc-coordinating amino acids and glutamic acid (Glu) residues that facilitate proton shuttling^[9]^.

In contrast, research has been limited on C-to-U editing and its significance in cancer^[10]^. The first and most important enzyme identified for C-to-U editing is APOBEC. It acts with RNAs as its cofactor. APOBEC1 principally deaminates Apob, which is important for lipoprotein metabolism among APOBEC family. Multiple members of the APOBEC3 (A3) family have been associated with lower antiviral defenses. Notably, A3G, a key target of cancer therapy, can further increase the tumor resistance to radiotherapy^[11,12]^.

In RNA editing experiments, commonly employed strategies involve utilizing endogenous ADAR enzymes or engineered ADAR recruitment (arRNA) to induce site-specific RNA editing. However, these traditional approaches exhibit notable limitations, including low editing efficiency, poor durability, and a high risk of off-target effects, where the editing tool unintentionally modifies off-target sites. Katrekar *et al* developed circular ADAR recruitment guide RNA (cadRNA)^[13]^, while Yi *et al* introduced covalently closed circular arRNA^[14]^. These innovative designs have improved the efficiency of RNA editing immensely. Moreover, Katrekar’s team has included interspersed loops in the antisense structure, while Yi’s group has removed uridine-adenosine pairing at off-target sites that have significantly lowered off-target damage. These advances allow for more precise and efficient RNA editing, showing wide application potential in the field of cancer treatment and expected to usher in new breakthroughs in cancer research^[15]^.

This review aims to present a comprehensive overview of RNA editing in cancer treatment. The historical journey of RNA editing research is overviewed initially, followed by the identification of molecular mechanisms involved in RNA editing and their significance in cancer are extensively discussed. Reviewing the preclinical studies aims at exploring the therapeutic potential of RNA editing. New RNA editing tools and other hot spots receive special emphasis. Also covered is the RNA editing studies, technical challenges, and research future directions to help clarify its clinical translation pathway. While recent reviews have described many molecular mechanisms of RNA editing, here we focus on their clinical translation and provide an account of the great promise of this double-edged sword in surgical treatment. Through this appointment, an initiative has begun that connects RNA editing and clinical surgical oncology. The ultimate goal is to explain how RNA editing can transform new anticancer strategies and making effective and precise cancer therapies that advance personalized cancer therapy for better clinical outcomes.

Literature Search Strategy: For this review, literature retrieval was conducted through the PubMed database using Boolean logical operators. Key search phrases included “RNA editing,” “cancer,” “ADAR,” “APOBEC,” “CRISPR-Cas13,” “drug resistance,” “off-target effects” etc. In this study, we focused on the research related to the mechanisms, associations, tools, and clinical application of RNA editing in cancer. We excluded any article that was not in line with the pre-decided theme, had bad quality, or did not talk about key technologies. The included articles were selected from the year 1986 till April 2025, preclinical papers and review articles published in a decade (2015–2025) were prioritized.

HIGHLIGHTS

- RNA editing, a post-transcriptional modification mechanism, has a long and evolving research history, showcasing diverse functions in cancer. It serves a double purpose, and under some circumstances, it stimulates tumor progression, whereas in others, it has tumor-suppressive effects.
- Recent advancements in RNA editing tools, particularly CRISPR-Cas13 systems and SNAP-based platforms, demonstrate significant potential in cancer therapy. Nonetheless, these technologies also faced issues such as off-target effects and the need for accurate dosage administration.
- RNA editing has the potential to modulate oncogene expression, repair mutated genes, and enhance cancer immunotherapy. Nonetheless, these effects on tumors are complex yet quite the opposite in normal biological processes.
- Although RNA editing holds significant therapeutic potential in oncology, it faces challenges related to precise targeting, safety, functional elucidation, and the optimization of editing efficiency. Research in the future must focus on them to enable clinical translation.
- From the perspective of surgical oncology, by leveraging the dual roles of RNA editing and referring to various pathways of RNA editing in the field of tumor molecular biology, this article integrates and proposes that RNA editing can be applied to various perioperative decisions, prognostic prediction, as well as the development of novel adjuvant or combined treatment regimens.

It is worth noting that any artificial intelligence tools were not used in any stage of this review including literature retrieval, data integration, figure and table design, and manuscript writing. The TITAN checklist provides a comprehensive framework for declaring and documenting Al applications throughout the manuscript, ensuring clarity and reproducibility (https://doi.org/10.70389/PJS.100082)^[16]^.

## Historical perspectives on RNA editing

The term “RNA editing” was coined in the late 1980s by Benne *et al* to describe the insertion and deletion of mRNA in Trypanosoma mitochondria^[1]^. Initial research already associated RNA modifying to a variety of mammalian diseases. For example, the dsRNA-adenosine deaminase mediates hepatitis B virus antigenome modifying. Also, place-specific nuclear RNA modifying, which regulates channel desensitization speed, has a connection with neurodegenerative diseases^[17,18]^. The results obtained from these findings have inspired significant follow-up research investigation on the diseases and therapeutic approaches associated with RNA editing.

**Figure 1.**
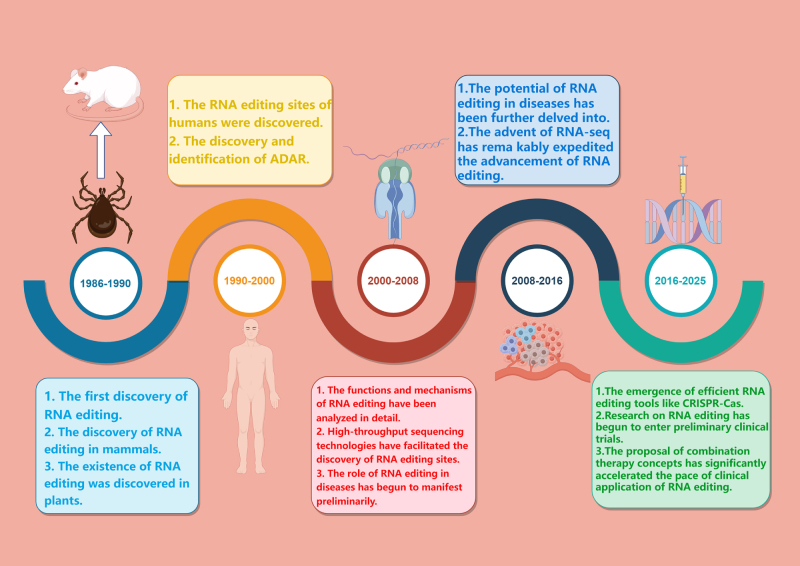
Historical progress on RNA editing. This timeline outlines the key milestones in RNA editing research, from fundamental discoveries to clinical translation, highlighting its emerging potential in cancer treatment. The development is categorized into five stages based on technological breakthroughs. This figure presents the mechanisms of RNA editing, beginning with an initial elucidation of these processes as RNA editing the transition from microorganisms to mammals. It presents the technological expansion and the advancement of human-focused studies, particularly in identifying subtypes of the ADAR enzyme. Furthermore, it presents a functional analysis that clarifies editing mechanisms and expands the identification of editing sites through high-throughput sequencing technology, along with early demonstrations of RNA editing relevance to oncology. There is an increasing body of evidence correlating RNA editing with tumorigenesis. Last, the potential for clinical application is presented, emphasizing the emergence of various new RNA editing tools and the integration of multi-omics data to support precision treatment (By Figdraw).

The advances of 21st-century next-generation sequencing technologies focused the RNA editing research toward therapeutic applications. The clustered regularly interspaced short palindromic repeats (CRISPR)-Cas system is an adaptive protein complex present in most bacteria and almost all archaea to protect against invasions of viruses and foreign objects. The influence of CRISPR on RNA was discovered back in 2006 by Kira S. Makarova and co-authors, who proposed a putative mechanism of action^[19]^. Significantly, the group of Alexandra East-Seletsky characterized the biochemical properties of Cas13 in 2016 and confirmed it possesses strong RNA-cleaving activity^[20,21]^. In 2017, fusion of Cas13 and ADAR allowed for taking RNA editing to the next level. Ever since the development of more precise RNA-editing tools, RNA editing has made significant strides in the application for disease therapy^[22]^. At present, various constraints mean that there are no clinical trials underway for RNA editing and cancer. However, the clinical trial of a non-oncological therapeutic drug based on RNA editing for the treatment of α-1 antitrypsin deficiency was started in 2023 (NCT06186492). RNA editing-based therapy has taken a real start with this important milestone. With further developments and improvements, RNA editing may present a great opportunity for curing cancer patients in the future^[23]^ (Key milestones of RNA editing are illustrated in Fig. 1).

At present, the blending of research into disease pathogenesis with the analysis of protein mechanisms is accelerating the progress of RNA editing toward therapeutic applications. RNA editing technologies are moving closer to clinical translation through improved delivery systems and editing specificity. Through continuous progress, if RNA editing can effectively address the daunting issues of target specificity, drug resistance and the initiation and termination of RNA editing, it will be in a good position to provide pioneering solutions for the precision intervention of major diseases including inherited diseases and malignant tumor.

## Mechanism of RNA editing

RNA editing refers to any site-specific modification within RNA molecules. In particular, RNA editing often implies a post-transcriptional modification that is catalyzed by adenosine deaminases or cytidine deaminases. At the specific focus of this article are cancer-related mechanisms. The process of RNA editing mainly implicates two key enzymes ADAR (A-to-I) and APOBEC (C-to-U). These modifications predominantly occur in non-coding regions, including introns, non-coding RNAs, and the 3’ untranslated region (UTR)^[24,25]^.

### Mechanisms of ADAR enzymes in RNA editing

The ADAR family in humans consists mainly of three members: ADAR1, ADAR2, and ADAR3. ADAR1 is widespread, while ADAR2 is mainly found in the brain and arteries, according to studies. In contrast, ADAR3 lacks RNA editing activity^[26,27]^. ADAR1 and ADAR2 perform A-to-I conversions mainly through hydrolytic deamination^[28,29]^. Substitutions in the encoded protein often occur as a result of translation when inosine is recognized as guanosine^[30]^. ADAR enzymes contain a number of double-stranded RNA binding domains (dsRBDs) which promote specificity for dsRNA^[31]^. One dsRBD situated proximal to the C-terminus of ADAR does not evolve in any way during catalysis^[32]^. Studies suggest that this particular domain may be critical for the catalytic center formation of ADAR enzyme, showing significant importance^[8,33]^.

#### Mechanism of the ADAR1 enzyme

**Figure 2.**
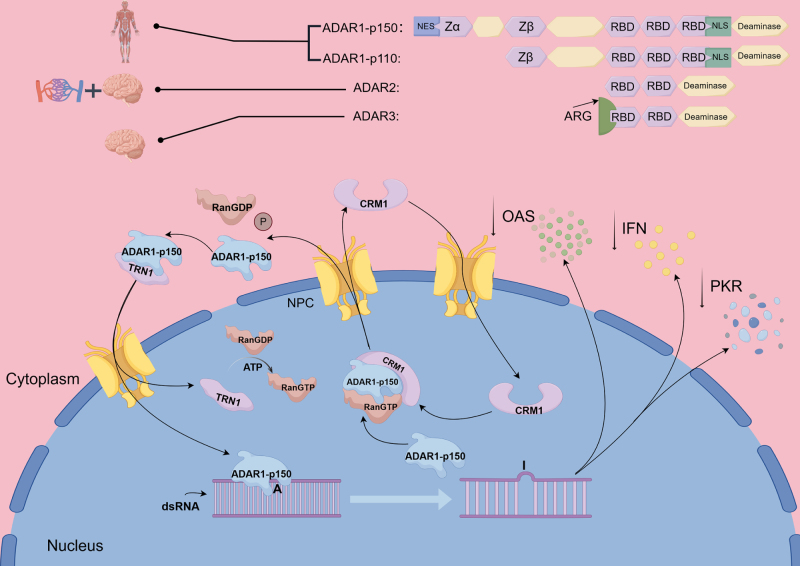
Domain architecture of the ADAR enzyme family and cellular localization/functional mechanisms of ADAR1-p150. This figure comprehensively depicts the domain structures and tissue distribution of human ADAR enzymes, using ADAR1-p150 as a representative model to illustrate cellular localization and functional mechanisms. ADAR1-p150 contains a nuclear export sequence (NES), Zα and Zβ domains, two double-stranded RBDs, a nuclear localization sequence (NLS), and a deaminase catalytic domain, which collectively facilitate nucleocytoplasmic shuttling. In contrast, ADAR1-p110 lacks the NES but retains the Zβ domain, RBDs, NLS, and catalytic domain, leading to its predominant localization in the nucleus. ADAR1 is ubiquitously expressed across various human tissues. In comparison, ADAR2, which comprises two RBDs and a catalytic domain, is primarily expressed in the brain and arterial vasculature, where it plays a crucial role in regulating neurological functions. ADAR3 contains an arginine-rich domain (ARG), one RBD, and a catalytic domain, with its expression predominantly observed in the brain. ADAR1-p150 undergoes dynamic CRM1-dependent nucleocytoplasmic trafficking, which involved three key steps: Nuclear Export: In the nucleus, ADAR1-p150 to RNA and CRM1, facilitating exportin-mediated translocation to the cytoplasm. In the cytoplasm, the hydrolysis of RanGTP hydrolysis releases ADAR1-p150, allowing it to bind to target dsRNA and catalyze A-to-I editing. Subsequently, cytoplasmic ADAR1-p150 interacts with TRN1 for re-import into the nucleus, where it dissociates to participate in nuclear RNA editing. The edited dsRNA suppresses MDA5-MAVS signaling pathways, attenuating type I IFN responses and protein kinase R (PKR) activation, thereby maintaining immune tolerance (By Figdraw).

ADAR1 has two isoforms, ADAR1p110 and ADAR1p150 which both have three dsRNA binding domains. ADAR1p110 possesses one nuclear localization sequence (NLS), which localizes it to nucleus. In contrast, ADAR1p150 contains both an NLS and a nuclear export sequence, allowing it to be found in both the nucleus and the cytoplasm (Fig. 2 describes the details of ADAR1 nuclear shuttling, and distribution sites and key structures of the ADAR family in the human body)^[30,34]^.

ADAR1p110 is important in the stress response as it binds to endogenous dsRNA and competitively inhibits Staufen1-mediated mRNA decay^[30,35]^. In contrast to the earlier findings, recent studies emphasize the role of ADAR1p150 in regulating immune signaling pathways including MDA5-MAVS^[36]^. The removal of ADAR1 leads to embryonic death in mice^[37]^, and so far, there have been no reports of live births with a complete deficiency in humans. This means that ADAR1 probably has a similar role in human embryonic development.

During RNA editing, the A-to-I conversion is facilitated by ADAR enzymes via the hydrolytic deanimation of the C6 position of adenosine to form inosine. ADAR enzymes mostly function at dsRNA regions that are longer than 20 base pairs in case of RNA editing sites. Sequencing technologies show that the majority of editing sites are located in dsRNA structures containing Alu that reside within introns and 3’ UTRs. The introduction of new nucleotides through RNA editing can cause splicing machinery misrecognition during mRNA maturation leading to the exaptation (exonization) of intronic Alu. RNA editing may therefore cause the introduction of new amino acids into expressed proteins and result in premature termination or extension of translation. However, the cellular systems do not aid in this editing. Endonuclease V is an intrinsic regulatory factor that can cleave Alu dsRNA and reduce its levels^[38,39]^.

Moreover, ADAR1 is capable of editing microRNAs (miRNAs). The Drosha-DGCR8 complex consists of several proteins that cut primary miRNAs into pre-miRNAs. They act together as a complex. After that, these pre-miRNAs are exported to cytoplasm by XPO5. Next, the Dicer-TRBP complex cleaves them into mature miRNA duplexes. The complex of miRNA with Argonaute (AGO) protein is the central component of RNA induced silencing complex, that is, RISC. This complex finally recognizes the 3’ UTR of target mRNAs to inhibit translation or ultimately degrade mRNAs. miRNA maturation can be subjected to RNA editing at any stage, including Drosha cleavage, Dicer cleavage, RISC loading, and target selection. The editing mainly causes the Drosha-DGCR8 and Dicer-TRBP complexes to be less effective in cutting. It also helps endonuclease V to find and destroy the edited miRNAs^[38]^.

#### Mechanism of the ADAR2 enzyme

ADAR2 has two forms which are ADAR2a and ADAR2b. ADAR2a is the full-length form, whereas ADAR2b lacks one or more domains^[40]^. ADAR2 is mainly localized in the nuclear compartment by a dual regulatory mechanism involving its N-terminal NLS and two karyopherin subunits [α1(KPNA1) and α3(KPNA3)] in the nuclear transport machinery. Importantly, E3 ubiquitin ligase WWP2 prevents cytoplasmic retention of ADAR2. This stimulates ubiquitination-mediated degradation of ADAR2 in the cytoplasm. As a result, ADAR2 levels are low in both the cytoplasm under physiological conditions^[38,41,42]^. ADAR2, unlike ADAR1, is mainly expressed in the brain and edits mostly coding regions, with a notable preference for intronic regions and affecting the function of proteins^[26]^. ADAR2 edits the GluA2 protein, which changes the ion permeability of AMPA receptors in motor neurons, and is thought to play a role in neurodegeneration^[43]^. ADAR2 binds to structures involving RNA and DNA. RNA hybrids prevent DNA repair and hinder the resection of DNA ends^[44]^. The main mechanism of ADAR2 is to facilitate the dissolution of RNA: DNA hybrids through RNA editing, which, in turn, promotes DNA end resection efficiently. In response to DNA damage, the core kinase of the checkpoint activates nuclear-localized ADAR2. When ADAR2 is activated, it binds directly to the RNA strand in the RNA: DNA hybrid, promoting the A-to-I conversion. This conversion creates mismatches in the intermediate strand of the RNA: DNA hybrid, which disrupts base-pairing between RNA and DNA. Any weakening must occur before the hybrid is broken down. Thus, ADAR2 RNA editing activity is an important contributor to genomic stability^[45]^. Though these vary, the mechanistic details of ADAR2 enzyme are similar to those of ADAR1 and are not elucidated further.

### Mechanisms of APOBEC enzymes in RNA editing

Enzymes APOBEC1, APOBEC2, and APOBEC3 are members of the APOBEC family. APOBEC1 is primarily found in cells of the intestine. APOBEC2 is seen mainly in cardiac and skeletal muscles. Similarly, APOBEC3 is linked to carcinogenesis and viral mutagenesis^[46,47]^. Like ADAR enzymes, the catalytically active domains and subcellular localization elements of APOBEC enzymes can also be recognized. In particular, the nuclear export mechanisms and localization signals of APOBEC1 allow it to shuttle between the nucleus and the cytoplasm. On the contrary, APOBEC2 is unable to actively translocate into and out of the nucleus because it does not possess localization signal sequences. APOBEC3 comes in two forms: a smaller single-domain form that diffuses freely and a larger double-domain form that does not diffuse into the nucleus because of its large molecular weight^[48,49]^.

#### Mechanism of the APOBEC1 enzyme

In humans, the sole RNA target of APOBEC1 that is established is that of APOB mRNA^[49]^. The editing process of APOB mRNA leads to the creation of two distinct protein isoforms. The first isoform is a full-length APOB protein responsible for cholesterol transport. The second and shorter of the two is known as short APOB. This shorter protein is known to primarily aid in transporting triglycerides. The isoforms are involved in cholesterol metabolism Moreover, APOBEC1 has been linked to tumorigenesis through its deaminase activity on DNA^[50,51]^. Notably, APOBEC1 does not have an elevated affinity for RNA, meaning it needs the RNA-binding protein APOBEC1 Complementation Factor (A1CF) to achieve optimal RNA editing activity^[52]^.

#### Mechanism of the APOBEC2 enzyme

Research on APOBEC2 remains limited. According to a recent study, binding to chromatin helps the APOBEC2 to facilitate muscle cell differentiation. However, no deaminase activity was identified, suggesting that it may have lost its original RNA editing activity^[53,54]^.

#### Mechanism of the APOBEC3 enzyme

**Figure 3.**
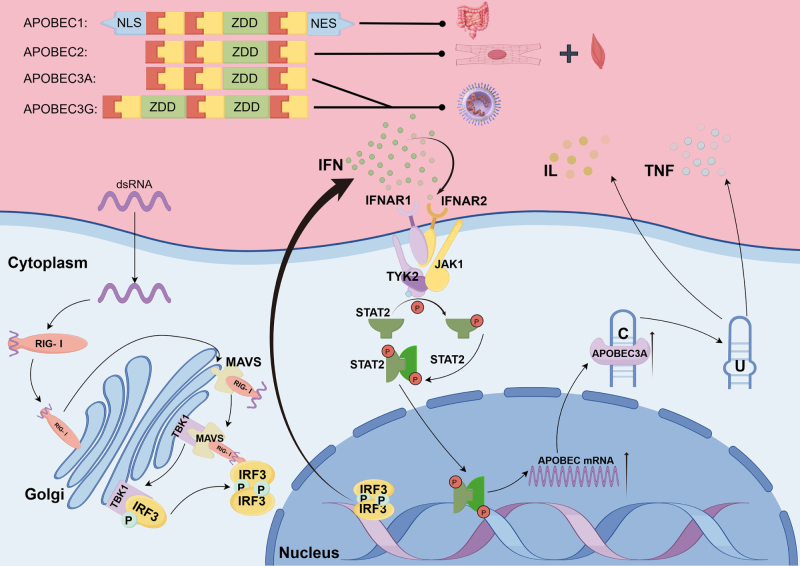
Domain architecture of the APOBEC enzyme family and the mechanistic basis of APOBEC3A activity. This figure offers a comprehensive illustration of the domain architectures of the APOBEC enzyme family and their tissue distribution in humans, using APOBEC3A as a representative example to demonstrate the RNA editing mechanism and the functional consequences triggered by exogenous dsRNA. APOBEC1 contains nuclear export and localization sequences, as well as a ZDD responsible for C-to-U editing. In contrast, APOBEC2 and APOBEC3A each possess a single ZDD, while APOBEC3G harbors two ZDDs. Upon entering cells, exogenous viral dsRNA activated the host pattern recognition receptor RIG-I, which recruits mitochondrial antiviral signaling protein (MAVS) to activate TANK-binding kinase 1 (TBK1). Activated TBK1 phosphorylates interferon regulatory factor 3 (IRF3), which allows phosphorylated IRF3 to dimerize and translocate into the nucleus. In the nucleus, it binds to IFN promoters, thereby initiating the secretion of type I IFN. Secreted IFN activates IFNAR1/IFNAR2 receptors on the cell membrane, facilitating the formation of a JAK1-TYK2 signaling complex. This process leads to the phosphorylation of STAT2, which subsequently dimerizes and translocates into the nucleus to activate the transcription of APOBEC3A mRNA. Elevated levels of APOBEC3A in immune cells enhance the synthesis and release of cytokines, including interleukins (ILs) and tumor necrosis factor (TNF), ultimately exerting antiviral effects (By Figdraw).

The critical activity of the APOBEC3 family of enzymes in host defense against viruses and virus-related disease is complex. Research has confirmed that it makes a major contribution to RNA editing^[55]^. The human APOBEC3 family consists of seven unique subtypes, namely A3A, A3B, A3C, A3D, A3F, A3G, and A3H. Similarly, editing of single-stranded viral RNAs, including HIV, by A3A has generated considerable interest. Additionally, A3A can regulate proteins such as amyloid precursor protein, which can facilitate cytidine deamination and the regulation of diverse microenvironments^[55,56]^ (Fig. 3 illustrates the specific mechanisms by which APOBEC3 participates in regulating the tumor microenvironment, as well as the main distribution sites and key structures of the APOBEC family in the human body). A3A mostly found in cytoplasm and brings about RNA editing at EVI2B and other sites. It shows a preference for stem-loop RNA elements. It has been suggested that editing at the tetracyclic sites occurs more efficiently on CAUC, then CUUC, then UAUC (target C). The first stem base pair has a substantial influence on editing efficiency^[57]^.

A3G is another functionally significant subtype. In mammalian cells, A3G exists exclusively in the cytoplasm in two forms, low molecular mass complexes and RNA bound high molecular mass complexes^[58]^. Studies involving cytokine stimulation reveal that TNF-α specifically causes LMM complex formation, while IL-1, IL-15, and IL-7 give rise to HMM complex formation^[58,59]^. Studies of structure show that the cytoplasmic HMM complexes contain mainly A3G that is connected to cellular RNA and cellular proteins through intermolecular RNA bridges. Zinc-dependent cytidine or deoxycytidine deaminase domain (ZDD; probably N-terminal) is the main target of these molecular bridges^[56]^. Evidence from biochemistry shows that treating HMM complexes with RNase releases A3G in enzymatically active form^[60]^. In contrast, A3G’s catalytic activity is inhibited by RNA binding. The detailed molecular mechanism for this inhibition has still not been clearly defined. Research suggests that A3G binds to viral components first before taking them to be ubiquitinated and degraded by the proteasome^[61]^. In addition, A3G can enter retroviruses and induce hypermutation of the negative-strand DNA, which causes G-to-A mutations during positive-strand synthesis^[56]^.

The polymorphic structures of both the ADAR and APOBEC families lead to variability in regulatory pathways. Any dysregulation of pathways involving any of these genes can lead to diseases like cancer. Moreover, the immune system development and maintenance are influenced by the ADAR and APOBEC enzymes, notably. To promote therapeutic applications of RNA editing, there is a need to understand their molecular mechanisms. By utilizing the relevant pathways, targeted selected strategies can be developed for the treatment and prevention of diseases^[13,14]^. Harnessing and modulating these pathways opens an interesting prospect to rectify abnormal RNA editing events which could ameliorate the disease pathology and obviate clinical benefit.

## The role of RNA editing in cancer

RNA editing can drive tumor initiation and progression via two major mechanisms. According to molecular studies, it modifies the RNA sites related to the cancer, so it modulates the dynamic expression of key oncogenes involved in malignant transformation, proliferation, and metastasis^[62]^. At the microenvironment level, RNA editing can alter the tumor immune microenvironment and impact host immune surveillance. RNA editing can either promote or suppress tumorigenesis by fixing pathogenic mutations. The complex role in cancer biology is evident from this bidirectional effect. By effectively using both functions, it may help slow down the progression of tumor in patients undergoing elective surgery and may also create a better environment inside the body and optimum surgical method for cancer patients during the perioperative phase. Thus, it is important to elaborate on the specific regulatory networks of RNA editing in different tumor types and their dynamic balance mechanisms so as to enhance RNA editing to truly play an effective surgical therapeutic role in various tumors. Table 1 summarizes the detailed mechanisms and functional roles (pro-tumor or anti-tumor) of RNA editing in cancer types. In the follow-up sections, we will discuss how RNA editing plays a role in drug resistance, invasion, and other tumor suppressor genes by showing relevant snippets (see cases in Table 1).

**Table 1:** Systematic summary of dual functional roles and clinical implications of RNA editing across various cancer types.

Types of RNA editing	Cancer types	Target genes/RNA	Function direction (pro-tumor/anti-tumor)	Mechanisms	Functional impacts	Clinical implications	References
A-to-I	Colorectal cancer	COPA	Pro-tumor	Generates I164V isoform, disrupts ER homeostasis, and activates UPR	Promotes metastasis; poor prognosis	Potential therapeutic targets	^[63]^
A-to-I	Osteosarcoma	ADAR1, ADAR2, and EMP2	Pro-tumor	Disrupts miRNA-mRNA pairing, abolishing oncogene suppression	Promotes malignancy and tumorigenesis	Prognostic evaluation; potential therapeutic targets	^[64]^
A-to-I	Leukemia	ADAR1 and dsRNA	Pro-tumor	Edits dsRNA to evade MDA5 recognition and suppresses dsRNA sensing	Maintains self-renewal; enables immune evasion	Assessment of disease status and prognosis; investigation of drug resistance mechanisms	^[65]^
A-to-I	Lung cancer	ADAR1 and NEIL1	Pro-tumor	Increases NEIL1 editing rate	Promotes malignant growth, invasion, and metastasis	Aids in diagnosis and prognosis prediction; treatment targets	^[66]^
A-to-I	Esophageal cancer	ADAR2 and SLC22A3	Pro-tumor	ADAR2 editing reduces SLC22A3 inhibition of migration and invasion	Enhances invasiveness, metastatic potential, and pseudopodia formation	Aids in diagnosis and prognosis; guiding personalized treatment	^[67]^
A-to-I	Melanoma	ADAR2, DOCK2, and mRNA	Pro-tumor	Editing enhances DOCK2 stability; activates Rac/Akt/NF-κB signaling	Inhibits apoptosis; promotes tumorigenesis and progression	Potential therapeutic targets; prognostic biomarkers; overcoming drug resistance	^[68]^
A-to-I	Glioblastoma	ADAR1 and GM2A	Pro-tumor	Edits GM2A 3’UTR to maintain expression and stem cell properties	Promotes proliferation and self-renewal; maintains stem cell properties	Potential therapeutic targets; personalized and precision treatment	^[69]^
A-to-I	Oral cancer	ADAR1, NEIL1, miR-381, CDH1, and VIM	Pro-tumor	ADAR1 overexpression induces EMT marker changes and oncogenic miRNA expression	Accelerates EMT; maintains malignancy; promotes migration, invasion and proliferation	Potential therapeutic targets; disease evaluation and prognosis; guiding treatment	^[70]^
A-to-I	Cholangiocarcinoma	ADAR1 and BRCA2	Pro-tumor	Edits BRCA2 3’UTR, blocking miRNA binding and stabilizing transcript	Enhances DNA repair and promotes drug resistance	Prognosis prediction; therapeutic strategies targeting drug resistance	^[71]^
A-to-I	Prostate cancer	ADAR1 and AR	Pro-tumor	Edits AR transcripts, introducing gain-of-function mutations	Increases proliferation under low androgen and promotes aggressiveness	Therapeutic resistance strategies; precision guidance	^[72]^
A-to-I	Multiple myeloma	ADAR1 and GLI1	Pro-tumor	Edits GLI1 mRNA, enhancing transcription and activating Hh pathway	Promotes tumorigenic regeneration and stem-like properties; confers drug resistance	Prognostic risk stratification; therapeutic targets and overcome resistance	^[73]^
A-to-I	Ovarian cancer	ADAR1, IFN, and dsRNA	Pro-tumor	Edits mRNA to evade immune detection; reduces immunogenicity	Suppresses IFN signaling; dampens immune response; modulates TME; enhances synergy with DNMTi	Potential therapeutic targets; prognostic determinants; combination therapy	^[74]^
A-to-I	Non-small cell lung cancer	E2F3 and ADAR1	Pro-tumor	ADAR1 stabilizes E2F3 mRNA; activates Rad18	Enhances DNA repair; contributes to radio-resistance	Therapeutic target; prognostic improvement and target radio-resistance	^[75]^
A-to-I	Hepatocellular carcinoma	ADAR1, dsRNA, and PGRN	Pro-tumor	Edits dsRNA to prevent immune surveillance; PGRN-EGFR activation	Facilitates immune evasion; inhibits tumor growth in deficiency	Potential therapeutic target; immunotherapy; diagnostic biomarkers	^[76]^
A-to-I	Hepatocellular carcinoma	ADAR1, Keap1, and Nrf2	Pro-tumor	ADAR1 deficiency impairs antioxidant defense, increasing ROS; overexpression inhibits Keap1, stabilizes Nrf2	Modulates Keap1/Nrf2 to reduce ROS and promote cell survival; confers resistance to therapy	Tumor prognostic biomarkers; therapeutic target; drug resistance	^[77]^
A-to-I	Colorectal cancer	DDX58 and ADAR1	Pro-tumor	ADAR1 editing enables PTIR1 generation; PTIR1 activates UCHL5	Suppresses antigen presentation and MHC I; impairs T cell recognition	Potential therapeutic target; prognosis prediction; immune microenvironment modulation	^[78]^
A-to-I	B-cell lymphoma	MAVS and ADAR1	Pro-tumor	Edits MAVS 3’UTR, enhancing stability and translation; activates signaling pathways	Activates NF-κB/ISG signaling; suppresses antitumor immunity; promotes T cell exhaustion	Potential therapeutic target; prognosis prediction; facilitating immune evasion	^[79]^
A-to-I	Non-small cell lung cancer	miR-411–5p and ADAR2	Pro-tumor	Editing enables miR-411-5p to target MET and suppress expression; reduced ADAR2 diminishes this function	Suppresses MET; restores TKI sensitivity; promotes apoptosis	Potential therapeutic target; overcomes drug resistance	^[80]^
A-to-I	Intrahepatic cholangiocarcinoma	ADAR1 and KPC1	Pro-tumor	ADAR1 edits KPC1, impairing its function and enhancing NF-κB signaling	Loss of tumor suppressor function; enhances NF-κB signaling; promotes EMT, proliferation, migration and invasion	Potential therapeutic target; prognosis prediction; immune TME modulation	^[81]^
A-to-I	Malignant mesothelioma	ADAR2, DHFR, and FPGS	Pro-tumor	ADAR2 maintains DHFR expression via editing; depletion enhances chemosensitivity and immune response	Reduces nucleotide synthesis and proliferation; enhances chemosensitivity; modulates immune TME	Overcoming tumor drug resistance; potential therapeutic target; immune TME modulation	^[82]^
A-to-I	Bladder cancer	ADAR2 and circ_0001005	Pro-tumor	AR suppresses ADAR2, promoting circRNA generation and PD-L1 upregulation via ceRNA mechanism	Promotes circ_0001005 accumulation and PD-L1 expression; facilitates immune evasion	Prognostic biomarker; augments immunotherapy	^[83]^
A-to-I	Endometrial carcinoma	AZIN1, ADAR1, and dsRNA	Pro-tumor	ADAR1 edits AZIN1, stabilizing oncoproteins; suppresses dsRNA sensing and apoptosis	Stabilizes oncoproteins; promotes proliferation and malignancy; maintains cell survival	Prognostic biomarker; guides therapy; potential target	^[84]^
A-to-I	Breast cancer	ADAR1, ATM, and POLH	Pro-tumor	Edits 3’UTRs of DNA repair genes, affecting mRNA stability	Promotes proliferation, invasion, survival and adaptation; enriches transcriptomic diversity	Prognostic biomarker; therapeutic target; guides therapy	^[85]^
A-to-I	Pancreatic ductal adenocarcinoma	ADAR1 and dsRNA	Pro-tumor	m6A modification impairs ADAR1 binding, reducing editing; dsRNA accumulation activates RLR-MAVS pathway	Leads to dsRNA accumulation and IFN response; enhances antitumor immunity	Prognostic biomarkers; potential therapeutic target; TME remodeling	^[86]^
A-to-I	Pleomorphic glioblastoma	ADAR2, PHKA2, and mRNA	Anti-tumor	ADAR2 edits PHKA2 mRNA, reducing stability and promoting degradation	Downregulates PHKA2; suppresses glycolipid metabolism and proliferation	Suppresses tumor growth; therapeutic target; combination therapy	^[87]^
A-to-I	Primary effusion lymphoma	miR-K12-4-3p, ADAR1, and AJUBA	Anti-tumor	Edits viral pri-miRNA, reducing mature miRNA production; suppresses PKR/MDA5 signaling	Reduces immunostimulatory dsRNA; facilitates immune evasion	Potential therapeutic target; biomarkers; immunosuppressive TME	^[88]^
A-to-I	Acute myeloid leukemia	ADAR2, COPA, and COG3	Anti-tumor	ADAR2 edits COPA and COG3, Suppressing their pro-oncogenic functions	Reduces leukemia stem cell burden; promotes differentiation; suppresses leukemogenesis	Potential therapeutic target; enhances chemo sensitivity; guides therapy	^[89]^
A-to-I	Ovarian cancer	ADARB1 and AKT	Anti-tumor	Suppresses AKT phosphorylation	Suppresses proliferation and invasion; correlates with immune cell infiltration	Potential therapeutic target; TME modulation; prognostic indicator	^[90]^
A-to-I	Hepatocellular carcinoma	ADAR2 and COPA	Anti-tumor	Edits COPA to generate I164V isoform, reducing its stability	Suppresses PI3K/AKT/mTOR signaling; exerts tumor-suppressive effects	Prognostic biomarker; therapeutic target	^[91]^
C-to-U	Hepatocellular carcinoma	APOBEC1 and A1CF	Pro-tumor	Promotes steatosis and activates β-catenin signaling	Promotes HCC initiation and progression; poor prognosis	Prognostic assessment; guiding therapy	^[92]^
C-to-U	Pancreatic cancer	APOBEC3G and PTEN	Pro-tumor	Inactivates PTEN, activating Akt	Promotes tumorigenesis and anoikis resistance	Prevention of metastasis; therapeutic target	^[93]^
C-to-U	Renal cell carcinoma	APOBEC3C, NF-κB, and mRNA	Pro-tumor	Enhances NF-κB signaling by antagonizing miRNA suppression	Promotes tumor growth and progression; enhances stress resistance	Guiding personalized therapy; prognostic assessment	^[94]^
C-to-U	Cervical cancer	APOBEC3 and p53	Pro-tumor	Induces off-target DNA damage and impairs DNA repair; compromises p53 function	Promotes mutational evolution and heterogeneity	Potential therapeutic target; improving prognosis	^[50]^
C-to-U	Breast cancer	APOBEC3B, MHC-I, and PD-1	Anti-tumor	Induces anti-tumor immune response; activates CD4^+^ T cells	Suppresses tumor growth; remodels immune TME; enhances sensitivity to immunotherapy	Assessment of immunotherapy response; therapeutic target; guides therapy	^[95]^
C-to-U	Cervical cancer	APOBEC3A, MAPK, and PI3K	Anti-tumor	Induces DNA damage; suppresses PI3K/MAPK signaling	Suppresses migration, invasion and proliferation; promotes apoptosis	Prognostic biomarker; potential target	^[96]^

Red shading highlights anti-tumor events; green shading highlights pro-tumor events.

EMT, epithelial-mesenchymal transition.

### Regulation of tumor resistance by RNA editing

Developing drug resistance is a major obstacle to cancer therapy which adversely affects drug efficacy and patient outcome. New studies have shown that RNA editing plays a key role in drug resistance in cancer. ADAR1 which A-to-I editing of stearoyl-CoA desaturase (SCD1) 3’UTR markedly increases the stability of SCD1 mRNA increasing intracellular lipid droplet formation. This process, accordingly, relieves endoplasmic reticulum stress caused by chemotherapy, so cancer cells survive during chemotherapy process^[97,98]^. It has been demonstrated that inhibiting ADAR1 activity to prevent SCD1 editing can help to reduce chemosensitivity of tumor cells^[98]^. The expression of ADAR1 is induced via the interferon (IFN) and JAK/STAT signaling pathways in gastric cancer organoids that are resistant to 5-fluorouracil + cisplatin^[98]^. In an ADAR clinical relevance study, the Tin-Lok Wong team observed that patients with high expression of ADAR1 and SCD1 had significantly shorter overall survival than those with low expression; moreover, those with high ADAR1 expression had a worse prognosis. Furthermore, Pearson correlation analysis of ADAR1 mRNA expression and SCD1 editing has shown correlation of ADAR1 expression with SCD1 editing in subjects (*R*^2^ = 0.154, *P* < 0.001). Furthermore, in gastric cancer patients who underwent chemotherapy, ADAR1 expression levels were found to be lower in patients who responded to chemotherapeutic drugs than non-responders. A link between the ADAR1/SCD1 axis and drug resistance of tumors in gastric cancer patients has been suggested by this^[98]^. It is still unknown whether the ADAR1/SCD1 axis mitigates drug resistance in other cancer cell types. Researchers used a melanoma mouse model and CRISPR gene editing to perform a study on the effect of ADAR1-deficient tumor cells. They found that these cells have an enhanced inflammatory response that is mediated by MDA5. Furthermore, they also display increased sensitivity to at IFN-induced dsRNA. This response also engages CHROME-encoded protein kinase R, thereby improving immune checkpoint response and overcoming drug resistance to immunotherapy^[99]^. Thus, ADAR1 serves a protective function for tumor cells during chemotherapy, but since it is expressed in many normal tissues, just inhibiting ADAR is imprudent. As shown in Table 1, ADAR has both pro-tumor and anti-tumor characteristics. Simply blocking RNA editing enzymes to reduce tumor drug resistance may also remove their tumor-suppressive effects, potentially increasing a cancer’s chance of emerging, rather than decreasing it. A promising strategy for combating tumor drug resistance is selective targeting of certain downstream pathways or editing events^[29]^.

APOBEC enzymes have been implicated in drug resistance. Recent studies reveal that besides its RNA editing activity, the enzyme APOBEC similarly catalyzes cytosine deamination in a DNA strand leading to DNA damage. DNA damage drives genomic instability in tumor cells that leads to greater heterogeneity of tumor cells and also development of drug resistance. However, there is little evidence of a link between RNA editing by APOBEC and drug resistance it mediates^[47,100]^. Consequently, in the clinic, the RNA-editing action of the relevant APOBEC needs to be disturbed minimally for developing new applications targeting these enzymes for cancer drug resistance.

In therapeutic studies of RNA editing for cancer drug resistance, it is important not to just target the indiscriminate inhibition of RNA editing related enzymes. We must look into what causes cancer drug resistance and the side effects of RNA editing enzymes at the same time. It is more important to focus on the mechanisms and processes by which editing enzymes engender drug resistance rather than the enzymes themselves.

### RNA editing-mediated tumor invasion process

In addition to its involvement in drug resistance, RNA editing plays a crucial role in tumor invasiveness. RNA editing dysfunction is a major player in the process of tumorigenesis through its effects on splice site recognition. This disturbance can boost how invasive cancer cells become. In the process of translation, inosine is recognized as guanosine because edited adenine is not recognized leading to substitution of amino acids^[101,102]^. A recent study shows that in colorectal cancer, RNA editing activates RAS homolog family member Q by converting serine to aspartic acid which enhances the invasiveness of tumor cells. In the presence of a KRAS mutation, this effect is enhanced even further^[4]^.

CircRNAs are reported to play important roles in modulating the invasive property of tumors. In gastric cancer, the 3’UTR of p21 and Smad4 is targeted by miRNA-224 to suppress their expression and promote the invasiveness of gastric cancer. On the other hand, circMAPK1 served as a sponge for miRNA-224 to induce the expressions of p21 and Smad4 and reduce the invasiveness. Nevertheless, ADAR1-mediated A-to-I editing can inhibit circRNA biogenesis. The editing process often takes place in the region of inverted complementary matching, which is crucial to circRNA formation. ADAR1 regulates circMAPK1 expression to promote the invasive capability of gastric cancer cells. Moreover, ADAR1 can be downregulated by Smad4 in a negative feedback manner. Experimental evidence further indicates that higher ADAR1 expression is related to shorter patient survival^[103,104]^.

Another important target of RNA editing is antizyme inhibitor 1 (AZIN1), whose editing has been linked to increased invasiveness of multiple cancers including hepatocellular carcinoma, esophageal squamous cell carcinoma (ESCC), lung cancer, and gastric cancer, as detailed in Table 1^[105,106]^. During the translation of the AZIN1 transcript, an editing of A-to-I occurs at the residue 367 inside its β-strand 15 by ADAR1. This modification makes for a substitution of serine (S) for glycine (G). The protein AZIN1 is stabilized by this post-transcriptional alteration, leading to its overexpression. The edited AZIN1 engages with antizyme, which lessens its anti-tumor impact and increases tumor invasiveness. A lot of tumors rely on the same mechanism^[106,107]^. In a study of 92 patients with hepatocellular carcinoma, 66 of the tumor tissues (71.7%) displayed overexpression of ADAR1 (*P* < 0.001), and 43 of the tissues (47%) showed a significant decrease in ADAR2. Findings also showed that patients with ADAR1 overexpression and ADAR2 underexpression had a higher rate of tumor recurrences (67.5%, *P* = 0.004), and liver cirrhosis (82.1%, *P* = 0.016) and shorter disease-free survival (DFS) with 30.1 months, (*P* = 0.003) than those with only one or none of the abnormalities. In tumor migration and invasion assays, tissue cells with higher ADAR1 expression showed improved capabilities of migrating and invading. On the contrary, ADAR2-expressing transduced tumor cells displayed the opposite phenotype. It was confirmed that ADAR was an independent prognostic factor for DFS with a *P*-value of 0.025 via multivariate Cox analysis^[108]^. Furthermore, another investigation into hepatocellular carcinoma showed that decreased expression of ADAR2 was significantly in the increased risk of tumor recurrence and decreased DFS^[91]^. RNA editing is associated with clinical-pathological features of hepatocellular carcinoma and influence the tumoral invasion significantly. Given the significant impact of RNA editing on tumor invasive abilities, it is essential to inhibit particular events of RNA editing or downstream pathways for the efficient reduction of tumor cell invasion. Targeted treatments that are tailored to specific patient characteristics could be the way forward in precision oncology^[102,109]^.

### RNA editing affects tumor suppressor gene expression

As stated earlier, RNA editing performs a key role in the promotion of tumor drug resistance and invasiveness, which indicates a largely pro-tumor role. Still, it also causes tumor-suppressive effects by regulating the expression of tumor suppressor genes. Various cancers have seen the identification of ADAR2 as a tumor suppressor. One way involves ADAR2 editing of PODXL mRNA. The editing alters a histidine to an arginine at the residues which decrease the tumorigenicity of gastric cancer cells^[110]^. The overexpression of ADAR2 inhibit the growth of ESCC tumors by mediating A-to-I editing of IGFBP7. This editing event makes IGFBP7 resistant to matriptase proteolysis. Subsequently, IGFBP7 inhibits Akt signaling pathway and prevents the phosphorylation of apoptosis-related agonists, thereby promoting tumor cell apoptosis. In summary, ADAR2 facilitates tumor cell apoptosis through the Akt pathway mediated by the IGFBP7 protein^[111,112]^. This pathway editing has an important regulatory function in the death of tumor cells. Moreover, tumor cells that overexpress ADAR1 promote AZIN1-driven malignant progression. A total of 69 pairs of primary ESCC tumor tissue samples and matched paracancerous tissue samples were examined in this section. The editing level of AZIN1 was significantly higher in tumor tissues than in paracancerous tissues. This difference was statistically significant (*P* < 0.0001). Moreover, the editing of AZIN1 by ADAR1 causes amino acid changes, which enable ESCC cells with malignant properties. *In vitro* studies showed that AZIN1 edited ESCC cells exhibited higher growth rates, colony-forming abilities, migration abilities, and invasion abilities than AZIN1 wild-type ESCC cells. According to the *in vivo* experiment in mice, the edited AZIN1 group demonstrated a significantly faster tumor growth than that of the wild-type and control groups (*P* < 0.05). The findings show that ADAR1 is an important protease that regulates tumor malignancy progression^[107]^.

Patients diagnosed with an astrocytoma can suffer from the hyper-excitability of neurons, which ultimately harms the neurons^[113]^. Happily, ADAR2 inhibits astrocytoma proliferation by promoting expression of cell division cycle 14B^[114]^. The dephosphorylation of Skp2 at Ser64 by CDC14B leads to its degradation, which, in turn, stabilizes the tumor suppressor proteins p21 and p27^[115]^. In investigating the functional role of ADAR2, we conducted a comparative analysis of wild-type and catalytically inactive ADAR2. This analysis confirmed that ADAR2 promotes the expression of the CDC14B gene through A-to-I editing^[114]^. According to a recent study, the androgen receptor (AR) directly inhibits expression of ADAR2 by binding to its promoter. This causes an upregulatory action on hsa_circ_0084171 (circFNTA) which is a circular RNA. Thus, ADAR2 could play an intermediary role that ameliorates the consequences of AR. Additionally, ADAR2 promotes the expression of PD-L1 in NK cells through miRNA regulation, which enhances the tumor-killing ability of NK cells^[83,116]^. Table 1 shows that ADAR2 works against ADAR1 to affect tumor invasiveness.

ADAR2 works on the mRNAs of cancer-causing proteins of cells. It either destabilizes these cancer-causing mRNAs or modifies those of anticancer proteins to render them resistant to degradation. ADAR2 has the ability to inhibit tumor cells from becoming cancerous due to this characteristic. Moderately increasing ADAR2 levels in postoperative patients can be useful for controlling undetectable or metastatic microtumors in the body. Moreover, elevating ADAR2 expression exclusively in tumor cells or the tumor microenvironment could lead to reduced tumor growth or development, which may allow for more complete surgical removal or the optimal timing of surgery, respectively. Moreover, physician stratification based on grade of tumor can be assisted through preoperative assessment of ADAR2 activity and downstream edited targets (PODXL) in biopsy samples. This thus allows clearly establish the resection extent and the necessity for extensive lymph node dissection. The RNA activity of ADAR2 may offer an innovative modality to augment existing clinical cancer treatment regimens^[25,82]^.

### RNA editing remodels the tumor microenvironment

Tumor cells utilize RNA editing to change the TME in their favor, ultimately avoiding detection and destruction by the immune cells. The intrinsic cytotoxic capacity of immune cells within TME can be utilized to block immune evasion^[117,118]^. The crucial component that this mechanism relies upon for success is ADAR1, which modifies the dsRNA to prevent its recognition by the dsRNA sensors and inhibits type I IFN signaling. Knocking out ADAR1 causes increased levels of immunogenic dsRNA, activation of retinoic acidinducible gene protein I (RIG-I), and increased production of IFN-β. As a result, IFN-β will trigger the activation of IFN-stimulated genes (ISGs) which will create chemokines CCL5 and CXCL10 that will recruit immune cells to the TME. In a study of ESCC, it was found that the researchers explored the link between TME and the RNA editing levels. They found that CD8^+^ T cell score positively correlated with RNA editing status in cancer. In the low RNA editing group, the proportions of CD8^+^ tumor-infiltrating lymphocytes and PD-1 positivity among esophageal cancer patients were higher than those in the high RNA editing group, indicating that RNA editing is associated with the TME. Moreover, IFN-β stimulation of tumor cells with low ADAR1 editing levels markedly increased chemokine secretion and CD8^+^ T cell recruitment capacity, thus changing the TME^[119]^. Lower levels of ADAR1 are associated with better therapeutic response in patients with ESCC receiving immunotherapy, as demonstrated by clinical validation^[120]^. Research shows that ADAR1 knockout coupled with DNA methyltransferase inhibition greatly enhances immune activation^[74,121]^. Besides, knockout of ADAR2 has an effect on chemokine synthesis and the localization of immune cells that is similar to that of ADAR1 knockout^[82]^.

**Figure 4.**
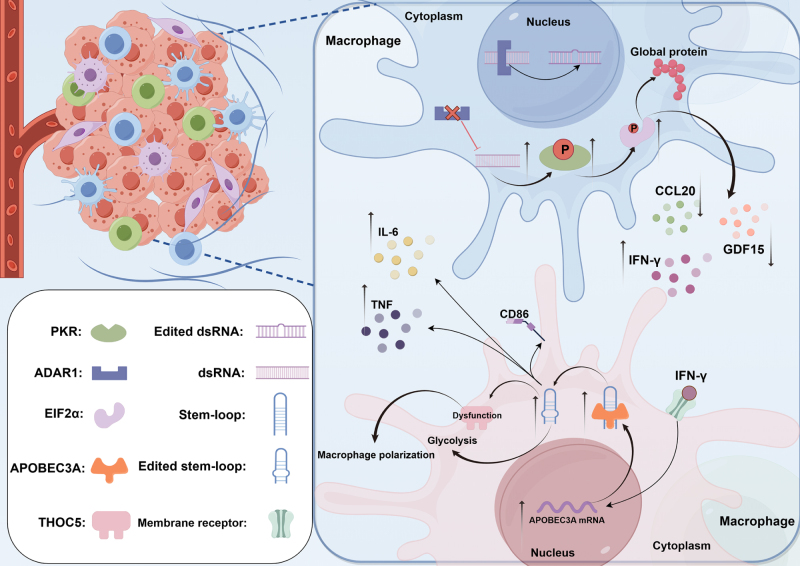
Role of RNA editing in macrophages within the tumor immune microenvironment. This figure illustrates the functional interplay between ADAR1 deficiency and elevated levels of APOBEC3A within macrophages in the tumor immune microenvironment. The deficiency of ADAR1 results in the cytoplasmic accumulation of endogenous dsRNA, which subsequently enhances the phosphorylation of PKR. Phosphorylated PKR stimulates eukaryotic initiation factor 2α (EIF2α) phosphorylation, resulting in widespread alterations in protein synthesis. These changes are associated with increased production of IFN-γ and a reduced expression of CCL20 and growth differentiation factor 15 (GDF15). The elevated levels of IFN-γ further upregulate the expression of APOBEC3A, which subsequently impairs the functionality of THOC5. These molecular events collectively drive macrophage polarization, augment CD86 synthesis, and elevate TNF production (By Figdraw).

The TME undergoes immune remodeling due to the activity of APOBEC enzymes. They encourage tumor cells to create and release a huge number of antigens, allowing T cell infiltration and activation. In addition, the diversity of T cell receptors is enhanced by the enzymes produced by the APOBEC family, leading to MHC-I upregulation and improvement of the capacity of immune cells to recognize and attack tumor cells. Moreover, the production of immune-suppressor cells is inhibited by APOBEC enzymes that maintains an active immune response^[95,122]^. Research indicates that APOBEC enzymes interact with the polycomb repressive complex 2, leading to a global decrease in abundance of H3K27me3, and specifically H3K27me3 molecular occupancy at the CCL2 promoter. This communication triggers the recruitment of myeloid-derived suppressor cells (MDSCs) and tumor-associated macrophages, which, in turn, promotes reprogramming of the TME^[123]^ (Fig. 4 elaborates on the molecular mechanisms and ultimate effects of the regulatory roles played by ADAR1 and APOBEC3 within the TME).

RNA editing can modify the immune microenvironment through two opposing mechanisms. The first mechanism is through enhancing immunogenicity, which can occur via the enhancement of antigen presentation and T cell activation mechanisms. The second mechanism is through interfering with immunosuppression, which can occur through limiting MDSC recruitment and inhibition of immunosuppressive signaling pathways. As compared to the non-specific cytotoxicity of conventional chemotherapy, the use of RNA editing to reprogram TME is more precise and has lesser systemic toxicity. This approach lays the molecular groundwork for designing immunotherapeutics that will activate the immune system and block immunosuppression specifically in tumors.

### RNA editing-mediated tumor metabolic reprogramming

Fresh evidence indicates that RNA editing can influence the energy metabolism and biosynthesis pathways of tumor cells by modulating the expression and activity of several enzymes. In the case of neuroblastoma, editing of the RNA, specifically the Q/R site of the glutamate receptor causes Ca^2+^ influx and thus metabolism of the cells^[124]^. In breast cancer cells, ADAR1-mediated RNA editing promotes METTL3 expression, a methyltransferase that catalyzes N6-methyladenosine (m6A) modifications at consensus RRACH motifs in RNA influencing RNA metabolism during tumor progression^[125]^. Telomere stability and genomic integrity also involve ADAR1-mediated editing of cellular RNA. ADAR1 inhibits transcriptional arrest and genomic instability via the inhibition of R-loop formation in a telomeric repeat region to maintain tumor cell metabolism^[126]^. Interestingly, APOBEC3 exhibits an opposing effect by promoting extensive DNA damage, which may contribute to tumorigenesis^[127]^. Another pertinent example is dihydrofolate reductase (DHFR), a crucial enzyme in the folate cycle. RNA editing enhances the stability and expression of DHFR mRNA, thereby facilitating the conversion of dihydrofolate to tetrahydrofolate, which supports nucleotide biosynthesis^[128]^. In the context of tetrahydrofolate, the RNA editing target site in DHFR is located within its 3’UTR, akin to that of SCD1, suggesting a potential preference or conserved characteristic in RNA editing targets within tumor cells.

### Regulation of cancer cell migration capacity by RNA editing

The migratory ability of tumor cells is closely linked to cancer prognosis and recurrence. RNA editing does complex things to regulate this process. Diverse and sometimes opposite features can be shown by the same RNA editing enzyme within a single cancer type. ADAR1, for example, can inhibit cancer cell migration by means of ARPIN^[129]^, while it enhances migration by regulating METTL3^[125]^. The cancer treatment is disrupted owing to this functional dichotomy. On the other hand, in other contexts, RNA editing is consistently pro-migratory. In lung adenocarcinoma, ADAR enzymes boost cell migration by increasing focal adhesion kinase stability or starting a certain R176G mutation in the RHOA subtype 2^[130,131]^. In thyroid cancer cells, ADAR1 promotes migration through editing of cyclin-dependent kinase 13 or miR200b^[132,133]^.

In addition to these effects of enzymes on themselves, evidence suggests a synergy among different RNA editing enzymes. Both ADAR and APOBEC enzymes were found to influence the migration of cervical cancer cells^[134,135]^. These findings underscore that RNA editing does not function through a singular, linear mechanism; rather, its roles in tumor migration are multifaceted, often interconnected, and contextually dependent. RNA editing can enhance tumor invasiveness, confer drug resistance, and induce the expression of tumor suppressor genes, while also being closely associated with the formation of the tumor microenvironment. From the surgical perspective, RNA editing-regulated tumor migration and invasiveness are directly related to the surgical margin selection and over- and undertreatment of patient’s management. The ability of RNA editing may alter these harmful behaviors of tumors, hence reducing the chances of over- and undertreatment.

Some editing events seem to be common to different cancers and editing enzymes, leading to similar phenotypic effects. This observation indicates that we should not focus on general inhibition of RNA editing or activation. Rather specific approaches with regard to site of editing and the downstream signaling pathways may inform optimum therapies with minimum side effects^[98,128]^. Moreover, the simultaneous use of RNA editing techniques and immune checkpoint blockade has yielded an enhanced antitumor impact beyond what either method can achieve^[25,136]^. This integrated treatment may represent a promising direction for future cancer therapies.

## New advances in RNA editing research

RNA editing refers to manipulation of transcribed RNA creating a valuable avenue for gene regulation as it requires modulation of the base sequences on transcribed RNA and not on the actual DNA. Moreover, RNA editing prevents damage of the genome. This has made RNA editing the center of investigation in a wide range of fields including cancer research. Through RNA editing, gene expression can be modulated on a post-transcriptional level by altering RNA transcripts. Due to an abundance of editing sites in the human transcriptome, RNA editing is likely to have considerable regulatory potential^[98]^.

Recently, Kumar *et al* reported CRISPR-Cas13, a tool for RNA editing and its use against retinal neovascularization, verifying its anti-VEGF potential^[137]^. Meanwhile, several RNA editing-based drugs have made their way into clinical trials, indicating that it is likely that these therapies may soon be available clinically. Table 2 summarizes the mechanisms, therapeutic potentials, developmental stages, and limitations of the most recent RNA editing systems to better guide researchers in selecting appropriate editing systems^[23]^.

**Table 2:** Emerging RNA editing technologies: research stage, mechanisms, therapeutic potential, and current limitations.

Latest therapeutic system	Mechanism	Target sites	Research stage	Key results	Therapeutic potential	Limitations	References
MAGE System	CasRx-mediated editing; PBAE29 delivery	Siglec-10 and SIRPa	Animal model and cell model	Enhances phagocytosis; reduces tumor volume	Targets tumor-associated macrophages	Immunogenic vectors; limited to abdominal tumors	^[138]^
RNCOs-D Technology	RNA nanosponges + Cas13a; EGFR aptamer	EGFRvIII mRNA and rRNA	Animal model and cell model	Cleaves mRNA; releases drug; and inhibits translation	Targeted therapy for EGFRvIII + tumors	Point mutations evade targeting; collateral effect is cell dependent	^[139]^
CREST Technology	CRISPR-Cas13 + RNA scaffolds; ADAR fusion	SMN2, KIF214, KRAS, and HBB	Cell model	Splicing and base editing; low off-target	Multi-target RNA correction; combos therapy	Variable efficiency; complex design	^[140]^
CRISPR-ADAReader System	Dual feedback RNA sensor; CRISPR-controlled	sesRNA, E2F1, and VEGF	Cell model	Activates apoptosis in tumor; spares normal cells	Precise RNA sensing and response	Viral vector risk; limited editing efficiency	^[141]^
REMORA Technology	Evolved rABE; fused with RBPs	ABE7.10, PUM1, and PUM2	Cell model	High-efficiency editing; records RBP binding sites	RBP mechanism analysis; dynamic recording	Complex setup; does not enrich binding sites	^[142]^
RESTORE Technology	ASO-recruited ADAR; RNA-DNA hybrid	STAT1-Tyr701 and SERPINA1	Cell model	Repairs mutation; reduces tissue damage	Edits inflammation/angiogenesis genes	Low efficiency; cell-type specific; interferon-dependent	^[143]^
SPRING System	Circular gRNA; hairpin-blocked aptamer	deadGFP, ACTIN, and TYMS	Cell model	High specificity; reduces mismatches	Precise editing for tumor genes	Sensitive to gRNA design; off-target not fully reduced	^[144]^

### Optimization of precision editing in CRISPR-Cas13 system

Cas13 is key protein of CRISPR-Cas like Cas12 and Cas9; it only edits RNA and not DNA. So far, three major variants of Cas13 have been discovered: Cas13a, Cas13b and Cas13c. Guided by CRISPR RNA (crRNA), Cas13a (also known as C2c2) binds and cleaves single-stranded RNA (ssRNA) from the target and collateral molecules^[145]^. The property has been exploited to develop new nucleic acid detection platforms^[146]^ and programmable RNA knockdown platforms^[22]^. Cas13b is highly efficient and specific for RNA knockdown. The site-specific A-to-I editing occurs when the inactivated Cas13b is fused with the domain of ADAR2^[147]^. This has led to the development of REPAIRv2, an optimized RNA editing system that exhibits significantly enhanced editing efficiency^[147]^. Despite its RNA-specific intent, REPAIRv2 may still influence DNA strands and produce off-target effects, indicating a need for further optimization prior to clinical application^[148]^. Recently, Yu *et al* introduced a light-inducible Cas13 system known as paCas13. Through light-dependent activation, this system facilitates precise spatiotemporal control of editing activity^[149]^. There is not much information available about Cas13c and its capability for RNA editing is not understood^[150]^, so it shall not be discussed further.

Even though Cas13 has been developed as a powerful RNA editing tool, the possibility of safety issues cannot be neglected^[151,152]^. Studies suggested that Cas13 has collateral activity in some human cells that degrade off-target non-viral transcripts, interfere with normal cell functions, and inhibit cell proliferation^[152,153]^. The Cas13 system must be integrated with robust monitoring strategies for off-target effects for clinical application. In that sense, molecular editing will facilitate tuning of editing efficiency and specificity.

### SNAP family of RNA editing tools

SNAP-ADAR is a designed RNA-editing tool that leverages a SNAP-tagged deaminase that is guided by a stabilized guide RNA. This device is highly efficient, accurate, and effective for long durations. Importantly, it offers the ability to control RNA editing yield quantitively, allowing for the halting of editing as per therapeutic requirements. This trait is especially useful in clinical cases^[154]^.

SNAP-ADAR can be coupled to either ADRA1 or ADRA2 or to a hyperactive variant. These conjugates exert different editing yields on some RNA target^[154]^. To improve the editing features of SNAP, another framework SNAP-CDAR-S has been initiated. SNAP-CDAR-S promotes C-to-U editing. This is accomplished by substituting the ADAR catalytic domain of SNAP-ADAR with a synthetic variant of ADAR2, which has been modified to carry out C-to-U editing^[155]^. The SNAP-CDAR-S has fewer bystander editing effects than the CRISPR-Cas13 system; however, the overall off-target rates were similar^[155]^. Even so, since SNAP-CDAR-S is derived from ADAR, some unintended editing from A-to-I occurs, indicating that it is not quite a precise C-to-U editing strategy. It is important for optimization to improve specificity and off-target effects in practical applications. In a recent study, Kumar *et al* showed that enhancing gRNA design can increase the efficiency, specificity, and outcome of SNAP RNA editing. The authors offered new methods to improve SNAP RNA editing even more^[156]^. The SNAP tool, like the Cas13 system, shows the duality of RNA-editing technology. Harnessing its therapeutic potential while limiting off-target effects will be an important goal as RNA editing advances toward the clinic.

### Development of precision RNA editing tools

For RNA editing to become an actual clinical therapy, there is an important necessity of precision in its editing activity. At present, the development of both the above tools faces the problem of huge off-target effects. Off-target effects occur mainly due to the RNA-binding domain binding non-specifically as well as deaminase domains constitutive activity inducing editing by ADAR. These off-target effects often result in transcriptional changes at sites outside the target gene, potentially triggering cancer cell evolution and limiting the clinical advancement of the RNA editing technology^[13,14]^.

The Guangye Li team has recently developed an RNA transformer adenosine base editor (RtABE) to enhance the specificity and efficiency of RNA editing considerably. Based on experience with minimizing off-target effects in DNA editing, the team hypothesized that the ADAR inhibitors (ADIs) could minimize off-target effects in the case of RNA editing^[157]^. They first screened the regulatory domains of the human APOBEC3 family (hA3A-hA3G) and found that the CDA1 domain of hA3D (A3DADI) could reduce off-target effects. Subsequently, the researchers generated a fusion protein comprising of the bacteriophage MS2 coat protein, ADAR2 deaminase domain (ADAR2DD), TSDD, which is an ADAR2 deaminase domain with a tobacco etches virus protease cleavage site, and A3DADI. The eagRNA_V5-mediated recombinant protease specifically cleaved the A3DADI domain from the fusion protein at the target site, which restores the RNA-editing deaminase activity of ADAR2DD. At the off-target sites, ADAR2DD remained constitutively inhibited in a state due to the lack of eagRNA_V5-mediated protease assembly and hence no off-target effects. To improve the specificity of their system further, the authors introduced mutations at the binding interface of ADAR2DD and dsRNA based on the crystal structure of this complex. Modifications of these types resulted in more robust recognition of target sequences and reduced binding of non-specific dsRNA. Compared to other editing tools, RtABE demonstrates higher editing efficiency with a lower off-target rate. The researchers also confirmed the therapeutic efficacy of the system in a mouse model of mucopolysaccharidosis type I *in vivo*, establishing safety, editing efficiency, and therapeutic effects of the RtABE system. Remarkably, there were no noticeable off-target effects on the entire transcriptome of the mouse model^[158]^. Nonetheless, the system is not without its difficulties. These challenges are presented by the complexity of the editor, the presence of non-human components that can elicit an immune response, and low editing efficiency at certain sites. While RtABE has certain limitations, it can still be a useful tool for providing valuable assistance in disease treatment in the future.

At the same time, Huiying Li’s team developed a photoactivatable A-to-I RNA base editor, a split ADAR2DD driven by the mini dCas13X and a light-controlled magnet system. Upon exposure to blue light, this editor is capable of assembling functional deaminases at defined sites, thus permitting single-nucleotide level regulation with low off-target activity. Despite this, editors may still be over-edited, with production effects gotten. The latent deaminases and the editor will be inactivated depending on their own half-lives. Enzymes and editors that are not inactivated in a timely manner can keep functioning and editing excessively. An accurate and efficient RNA editing system is needed to carry RNA editing from the lab to the clinic. Overcoming these inherent limitations is necessary for RNA editing to significantly contribute to cancer treatment^[159]^.

## Potential and prospects of RNA editing in surgical oncology

Cancer surgery may be augmented by a new approach utilizing RNA editing. Unlike classic chemotherapy, which has no ability to target, RNA editing acts on the post-transcriptional modification system by virtue of its high targeting ability and uses the body’s own regulatory system. In surgical oncology applications, this unique mode of action positions RNA editing as a new and very interesting tool.

RNA editing can modulate the expression of oncogenes by selectively editing the mRNA transcripts of these genes or related pathways. In the context of cervical cancer, A-to-I editing modifies amino acid residues within the YXXQ motif of bladder cancer-related proteins, thereby abrogating their suppressive effect on signal transducer and activator of transcription 3 (STAT3)^[160]^. Activation of STAT3 is an important event that aids in tumor development and progression^[161]^. Inhibition of A-to-I RNA editing may maintain the function of tumor suppressor proteins and exert antitumor effects^[6]^. RNA editing can help manage or reduce tumor size in an operative patient setting for tumor. It allows patients to meet the surgical criteria for clinical resection and offered more time to create a better environment for the procedure.

Editing of RNA may rectify the damages incurred from mutations or dysfunctional transcripts, restoring the normal functions of tumor-suppressor genes. Cox and colleagues created a new tool by binding a catalytically inactive PspCas13b with ADAR2 deaminase domain. The deaminase domain can correct base mutations that lead to a variety of diseases including cancer^[147]^. Gootenberg *et al* validated this conclusion through a nucleic acid detection platform known as SHERLOCK, utilizing HEK293 FT cells^[162]^. According to a comparison study carried out by Vogel *et al*, the SNAP-tagged ADAR2, under the exact and higher guidance of gRNA, has a superior editing efficiency and precision than Cas13b-ADARs system^[154]^. According to the findings, RNA editing can fix base mutations, which is advantageous. Due to this advantage, RNA editing can reduce inaccurate editing in mutant cells as well as reduce borderline tumor cells. It helps surgeons to minimize the scope of surgical resection, reduce the risks and complexity of surgery, and minimize the impact of surgery on the patients’ physical condition as much as possible. An extensive study and application of this natural benefit may be the basis for its clinical uses^[163]^.

RNA editing plays a crucial role in assisting surgeons with tumor immunotherapy. Immune checkpoint inhibitors, such as PD-1/PD-L1 antibodies, have significantly improved survival outcomes in certain cancer patients^[164]^. RNA editing enzymes alter immune responses by different mechanisms. For example, the expression of ISGs remains inhibited by ADAR1, which leads to immune evasion^[165]^, whereas deletion heightens tumor sensitivity to immunotherapy^[99]^. ADAR2, on the other hand, upregulates PD-L1 through miRNA signaling, thereby enhancing anti-tumor immunity^[83]^. Similarly, APOBEC3 promotes PD-L1 expression, which improves immunotherapeutic responses^[166,167]^. Furthermore, the excessive accumulation of dsRNA can initiate innate immune activation resulting in immune overactivation. ADAR1 functions to stabilize dsRNA, thus maintaining immune homeostasis^[168]^. Consequently, we expect that targeting a subset of RNA editing enzymes will boost the effectiveness of immunotherapy in surgical oncology, along with immune homeostasis. The combination of RNA editing with tumor immunology will alleviate the drug resistance and tumor cell escape from immunotherapy. Therefore, the drug dosage and the period of treatment will be as minimized as possible^[83,168]^.

RNA editing is linked with the resistance of tumor chemotherapy. RNA editing promotes cancer cells to overcome the repair mechanisms that target abnormal cells. This happens at the level of impaired immunity, increased tumor mutation rates, and epithelial-mesenchymal transition. The increased heterogeneity often leads to unpredictable drug resistance in tumor cells or allows them to survive via another signaling pathway during chemotherapy^[169]^. Notably, recent studies have suggested that the expression of ADAR1 may correlate with a good prognosis in oral cancer, although the mechanisms remain unclear^[170]^. This tissue-specific and context-dependent behavior underscores the necessity for personalized RNA editing-based interventions. The development of specific inhibitors targeting oncogenic editing events and the use of RNA editing to reshape the tumor immune microenvironment represent important directions for future studies Table 2 summarizes the considerable potential of novel RNA editing systems in cancer therapy as well as their limitations.

## Challenges of RNA editing and future options

RNA editing is an essential post-transcriptional gene regulation phenotype with the potential to maintain physiological homeostasis and which also reveals therapeutical potential in cancer. If we want to routinely apply RNA editing to clinical practice, there are several issues to be resolved. First, RNA editing affects a wide range of genes – from tumor suppressor genes and proto-oncogenes to the components of innate immunity. The broad spread of this influence is a strong signal for making precise targeted edits with fewer off-target effects. Unintentional editing may create havoc and threaten the safety and efficacy of products. High-throughput sequencing and editing strategies have advanced recently. Researchers are now introducing Cas13 into exogenous ADAR. Others are adding on an R/G or R/S structure to try and recruit endogenous ADAR. These all help reduce off-target effects^[143,171]^. Second, RNA editing, like DNA editing, has safety concerns. Despite the maturation of next-generation sequencing technology, studies indicate that false positive rates remain elevated in both experimental data and publicly available databases^[172]^. These databases are often used to annotate RNA editing sites, and it is important to be cognizant of their limitations and choose the right resource according to their specific research context. Table 3 list details of the widely used RNA editing databases, their unique contributions of each database, and assist the researchers making proper use of them based on their unique attributes during their study. It is crucial to reduce the false positive rate of RNA editing by integrating RNA editing tools into advanced systems. According to Tran *et al*, RNA Editing Detection Informatics Tool (REDIT) is a powerful algorithm that enhances the precision and credibility of RNA editing site detection^[173]^. Third, while RNA editing has increasingly been recognized as a prominent mechanism in the regulation of gene expression, its functional characterization remains a significant challenge. On one hand, capturing the dynamic changes of RNA editing in real time is difficult^[174]^; on the other hand, the biological significance of individual RNA editing events is often complex and context dependent. Fourth, more specifically, clinical applicability continues to be limited by editing efficiency and yield. Despite significant advancements through the use of tools like SNAP-labeled ADAR and CRISPR-Cas13 systems^[154,175]^, the present editing efficiency levels are not sufficient for clinical use. As such, both high and low levels of RNA editing can have negative effects^[34]^.

**Table 3:** Data types, species coverage, core features, search tools, and application limitations of cancer research-related RNA editing databases.

Database	Data type	Species	Unique features	Editing type	Search tools	Limitations	References
REIA	8.4 M A-to-I sites; multi-omics data	Human	Interactive multi-omics correlation; visualization; cross-cancer comparison	A-to-I	RESs Browser, Gene Browser, and Cancer Browser	Lacks genomic sequences and normal tissue data; unannotated novel peptides	^[176]^
LNCediting	LncRNA editing sites	Human, Rhesus, Mouse, and Fly	lncRNA structure prediction; miRNA-lncRNA interaction; cross-species comparison	A-to-I	Multi-dimensional search by site/lncRNA/miRNA	No APOBEC editing; limited to short lncRNAs; and computational predictions only	^[177]^
REDIportal	4.6 M A-to-I events; GTEx data	Human	Dynamic editing level visualization; tissue-specific filtering	A-to-I	Genomic coordinates, gene names, and tissue types	Limited disease samples; mostly polyA-enriched libraries	^[178]^
RADAR	1.3 M A-to-I sites; manual annotation	Human, Mouse, and Fly	Tissue-specific dynamics; evolutionary conservation; multi-source data integration	A-to-I	Genomic position, gene name, and repeat type	No dbSNP links; slow updates	^[179]^
DARNED	A-to-I and C-to-U; EST/SNP/miRNA data	Human	Non-redundant dataset; distinguishes editing from SNPs	A-to-I and C-to-U	Genomic scope, gene/region, and tissue origin	Limited APOBEC data; single species; and manual updates	^[180]^
dbRES	Experimentally verified A-to-I and C-to-U sites	Plants, animals, fungi, and viruses	Amino acid changes; BLAST-based search	A-to-I and C-to-U	Gene name, organism, and editing type	Low update efficiency; lacks functional annotations	^[181]^
e23D	RNA editing with 3D structure mapping	Human, Mouse, and Fly	3D visualization; pathogenic mutation overlay	A-to-I	Gene symbol, genomic interval, and editing level	Relies on external databases; some data are theoretical	^[182]^
GPEdit	edQTLs across 33 TCGA cancers	Human	Pan-cancer edQTL; survival/GWAS integration; drug response prediction	A-to-I	Cancer type, SNP ID, and genomic interval	No APOBEC data; European-only population; and no trans-edQTLs	^[183]^
REDH	Hematopoietic cell editing; mouse/human data	Human and Mouse	Hematopoietic-specific; differentiation/malignancy focus	A-to-I	Cell groups, disease types, and clinical parameters	Limited tumor types; no treatment outcome data	^[184]^
MiREDiBase	miRNA editing in primates	Human, Gorilla, Chimpanzee, and Rhesus macaque	miRNA-focused; evolutionary analysis; target impact prediction	A-to-I and C-to-U	Species, editing type, and genomic region	Low experimentally validated sites; limited APOBEC data	^[185]^

RNA editing technologies are advancing rapidly despite these challenges^[186]^. It is expected that in the future, RNA editing tools will be more specific, allowing for targeted modifications that occur at the site in a specified RNA^[186]^. The next generation of editing tools will showcase more diversity. They will be used to address more complex diseases. These will include diseases beyond neurodegenerative diseases and cancer^[172,187]^. Importantly, future RNA editing may incorporate a “stop button,” allowing for precise temporal control and minimizing the risk of excessive or prolonged editing^[65,188]^. Furthermore, DNA template-independent editing may allow multiple RNA targets to be modified and reduce genomic damage and enhancement of editing efficiency^[45]^. Concerning potential off-target effects of RNA editing, a new RNA editor, RtABE, achieves targeted editing by site-specific assembly of the editing enzymes. This strategy limits off-target effects and shows promise for new studies to refine targeted editing with greater accuracy. Understanding RNA editing can highly benefit from the integration of multi-omics data such as genomics, transcriptomics, and proteomics. This combination will provide insight into how RNA editing interacts with other processes in the cell in cancer^[189,190]^. A comprehensive research strategy will enable the development of personalized treatment strategies and, consequently, improve treatments using RNA editing. As the years go by and technological refinement takes place, tumor typing and malignancy are expected to be assessed preoperatively with RNA editing technology. Consequently, this will enhance the accuracy of decision-making by clinicians. While performing the surgery, RNA editing can help surgeons determine the surgical resection margins more accurately. Furthermore, it can help gauge the range of tumor infiltration in real-time and achieve R0 resection. Meanwhile, in nervous system tumors, the reversible RNA editing can manipulate genes related to neuronal excitability to better preserve neurological functionality. After surgery, RNA editing can assist in overcoming chemoresistance in tumor cells or reshaping the immune microenvironment to boost or regulate immune cell activity, extending tumor recurrence time. It also aims to destroy tumor cells that may have remained in blood vessels or lymphatic vessels after surgery to prevent tumor recurrence. Ultimately, through technologies like SCISSOR (Selective Cleavage and Intramolecular Stitching of RNA), RNA editing is expected to edit immune cells *in vitro* or create RNA editing-based vaccines. These vaccines can generate neoantigens by introducing frameshift mutations, eliciting patients’ own specific antitumor immune responses, which holds broad potential for clinical application^[191]^.

Despite the significant progress that RNA editing has achieved in areas such as cancer treatment, degenerative diseases, and vaccine development^[192,193]^, as well as its broad prospects, technical and safety-related challenges continue to pose key obstacles to its clinical implementation^[194]^. Future research directions should target specificity for editing, controllable editing systems, as well as combination therapeutic strategies^[195]^. To address the dual-edged nature of RNA editing in the future, three primary optimization directions have emerged. Traditionally, this includes improving system delivery to site-directed editing enzymes. Recent advances in research have brought several innovations. One strategy involves inhibiting enzyme systems, allowing editing enzymes to assemble only at desired locations, thereby silencing non-target sites^[158]^. A second technique is using light-controlled switch systems. In this case, light-guiding technology, for example, optical fibers, is embedded at the site of interest to directly control the initiation of editing via light on/off^[159]^. Continuous innovation and strict optimization in RNA editing may result in further development of clinical cancer therapy. However, this outcome is not assured. For instance, issues of non-therapeutic RNA editing in non-target tissues, limited targeting by classical RNA delivery platforms, and inactivation of editing enzymes after the reaction in recent studies indicate the need for balanced assessments. In the future, RNA editing applied in surgical treatment may mainly focus on a series of perioperative researches, including cancer localization and staging diagnosis, inhibition and/or reduction of tumor volume growth, and evaluation of postoperative prognosis. At the end, RNA editing could deliver precise and personalized treatment regimens. However, its actual capacity to provide therapeutic or surgical guidance within a clinical oncology context remains to be evidenced^[169,196]^.

## Discussion

RNA editing emerged as a frontier in cancer research, demonstrating significant potential for clinical translation. This review discusses the history of RNA editing. In the 20th century, scientists first started mechanistic studies on RNA editing. Over the years, RNA editing has evolved into a vital therapeutic tool. The present review describes the processes of RNA editing in detail with focus on the functions of the ADAR and APOBEC enzyme families. Furthermore, their broad and diverse roles in numerous disease contexts are also highlighted. In tumors, the multiple roles of RNA editing are highlighted, along with discussions about their relevance to drug resistance, tumor invasiveness, and immune evasion. Significantly, this review highlights that RNA editing can enhance cancer progression as well as display tumor-suppressor effects. RNA editing enzymes can have opposing effects in the same type of cancer, which is noteworthy. It is not only important to note the cancer types but also the relevant signaling pathways when it comes to clinical applications. Understanding all the pathways of RNA editing in tumors could help surgeons in the future to choose which RNA-editing enzymes with which targets. A lot of the original intention behind the introduction of RNA editing signaling pathways in this paper is to serve as references and inputs for the surgeon on whether to take up RNA editing adjuvant treatment modalities. The various functions of RNA editing encompass the entire cancer continuum, from initiation to treatment response and prognosis, highlighting its therapeutic importance. Besides, advances in RNA editing technology have led to new applications in nucleic acid detection and immune modulation, particularly because of COVID-19^[197,198]^.

This review addresses the new trends of RNA editing technologies. As a result of these developments the effective and accurate editing of edited versions must be regulated as excessive modification may lead to harmful effects. Also, the discussion describes the existing technical and safety difficulties while highlighting the potential for more future innovation. The editing of RNA molecules modifies the expression of genes after their transcription into RNA, without altering the coding sequence in the DNA. Thus, RNA editing could provide a significant edge for the engineering of personalized and targeted therapeutics. Thereafter, this article elaborates on the topic of RNA editing from the perspective of surgery in surgical oncology. It systematically elaborates the great potential of RNA editing from different sources for tumor staging diagnosis, benign and malignant tumor differentiation, and for surgical approach selection in the preoperative, intraoperative, and postoperative phases. However, the duality of RNA editing must also make physicians cautious about its use, as previously stated, particularly in light of the lack of clinical research on RNA editing in surgical oncology. Therefore, we hope that this review will encourage/guide the researchers to conduct relevant clinical studies which will enable RNA editing to serve clinical practice better. As the field evolves, interdisciplinary collaboration among molecular biologists, geneticists, oncologists, bioinformaticians, and clinicians would be essential to optimize the benefits of RNA editing in cancer therapy^[199]^. Future clinical strategies should incorporate RNA editing into personalized treatment regimens, preferably in conjunction with other modalities, to enhance therapeutic efficacy and improve patient outcomes.

RNA editing is a novel technology for gene engineering and part of genetic engineering. RNA editing is a separate category, even though it is similar to gene editing. In molecular biology, RNA editing refers to the addition of further information in RNA after it has been transcribed but does not alter the DNA coded RNA. With regard to genetics, this means that RNA editing alters the core coding of the genetic material in such a manner that there is little impact on heredity. On the other hand, approaches such as gene knockout, insertion, or substitution uses CRISPR/Cas9 and other systems to make permanent changes to the DNA, and these changes are passed along during cell division. RNA editing does not make permanent changes to the genome and, as a result, does not lead to gene pollution. This feature makes it very attractive for cancer therapy. In the past, RNA editing was thought to be adaptation forces in cancer biology. It has received less attention than it deserves. RNA editing should not be seen only as a regulatory mechanism. RNA editing has the potential to serve as a therapeutic target or therapeutic tool. However, the potential of RNA editing to reshape cancer treatment paradigms is undetermined. Many factors impede the development of RNA editing. For example, several factors, including the inherent safety of RNA editing, editing efficiency, the targeting specificity of delivery systems, off-target effects, and the initiation and termination of editing, present constraints on its development.

One of the major challenges in oncology is drug resistance. RNA editing enzymes are often implicated in the generation of tumor cell heterogeneity and tumor evolution. As evidenced by the targeted knockout of RNA editing enzymes incurring less drug resistance, such editing has a causal role. Thus, in surgical oncology treatment, RNA editing is likely to reduce your chemoresistance to tumor chemotherapy in the future, thus helping the patients to lessen their dosage and duration of chemotherapy after surgical resection. As of now, there is not enough substantial evidence to support this claim. In the future, we hope that more and more researchers will be engaged in clinical studies on RNA editing-related chemoresistance. This will help in optimizing the postoperative chemotherapy more quickly.

The functional modalities of RNA editing highlight the innate duality of RNA editing, which is attracting more and more attention. As noted in Table 1, the list of enzymes displayed in the left column shows that the pro-tumor and anti-tumor RNA editing capabilities are offered by the same ADAR for different cancers. For instance, while ADAR1 promotes tumorigenesis in lung cancer, it performs an anti-tumor role in primary effusion lymphoma. The contrasting roles show that duality in RNA editing’s nature and also how it works sharply differs in different tissues. These properties have somewhat impeded the clinical translation of RNA editing and imposed stringent requirements for RNA editing technology in clinical applications. Slight, moderate, and heavy editing can create totally different and even every type cancer treatment effects. This shows the need for control of the level as well as the time of editing consistent with precision oncology and personalized treatment. The clinicians involved in surgical oncology should monitor the amount and quality of RNA editing in the design or future conduct of clinical trials. This could be a reason why such experiments failed in some instances. Thus, one possible solution to this problem may be to research on corresponding antagonistic drugs that can act as an “editing switch” in the therapeutic development.

To sum up, RNA editing technology is an innovative value and research potential technology in cancer treatment. The current development of key technologies such as the CRISPR-Cas13 system and SNAP-ADAR technology, as well as the progressive emergence of high-precision base editors such as RtABE, hints at the future potential of RNA editing in molecular oncology. Simultaneously, RNA editing target and pathway diversity and duality in tumor molecular biology require surgeons to consider these. Surgeons will ultimately determine the final choice of application – and practical use – of RNA editing, even when it is fully developed as an adjunct surgical tool in the future. Doctors should make the final judgment on issues like dosage control, choice of specific targets, and balancing side effects and efficacy. It will be challenging to achieve the true surgical objective of “precision diagnosis and treatment” without understanding the molecular mechanisms of RNA editing. Future studies should aim to systematically elucidate, characterize, and the dynamic regulatory networks of RNA editing in tumorigenesis, progression, and drug resistance. In addition, it is necessary to pinpoint the interaction of RNA editing with other oncogenic mechanisms, including epigenetic regulation, tumor metabolic rewiring, and the tumor microenvironment. Through this manner, surgeons can avoid the off-target effects of RNA editing that causes iatrogenic tumor during the development or application of RNA editing in clinical practice, thereby unleashing the true therapeutic potential of RNA editing.

To promote clinical translation, there are four important scientific aspects to consider. First, a correlation model needs to be established between the tumor-specific RNA editing map and clinical stages to evaluate how RNA editing can regulate or be regulated by various tumor stages. Second, the dual role of RNA editing requires the development of fully controllable RNA editing tools, with an appropriate dynamic balance between targeted editing efficiency and off-target effects, which can be achieved using engineered ribozyme complexes. Third, based on multi-omics integration and Artificial Intelligence, a system for predicting individualized treatment responses and novel drug delivery mechanisms should be developed. The stability and targeting specificity of RNA editing can be improved greatly by novel drug delivery systems^[200,201]^. In the meantime, AI would help analyze the strengths and weaknesses of different treatment regimens, combine the findings, and formulate combination therapeutic strategies^[202]^. This method would enhance the formulation of precise treatment strategies and support the creation of combination treatment plans. Integrating current modalities such as CAR-T cell therapy and immune checkpoint inhibitors may enhance therapeutic efficacy through synergistic mechanisms. Last but not least, much emphasis must be placed on the safety RNA editing process. One example of the utility of conditional activation systems is limiting the action range of RNA editing enzymes to a controllable one. At the same time, there is a need for efficient and safe delivery systems that accurately deliver RNA editing tools to tumor cells. In surgical oncology, limited delivery methods such as a local injection during surgery or perfusion after surgery could minimize the risk of systemic exposure.

## Data Availability

Data sharing not applicable to this article as no datasets were generated or analyzed during the current study. All information is derived from publicly available articles and datasets.
